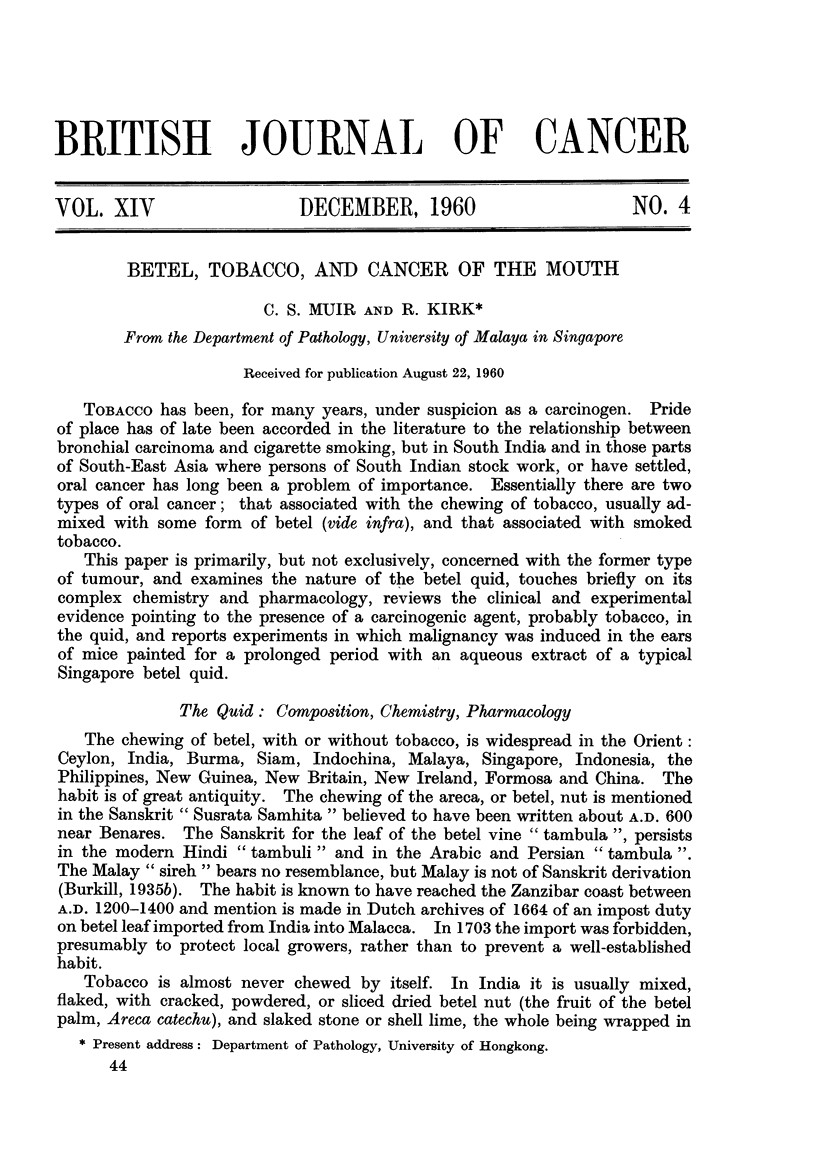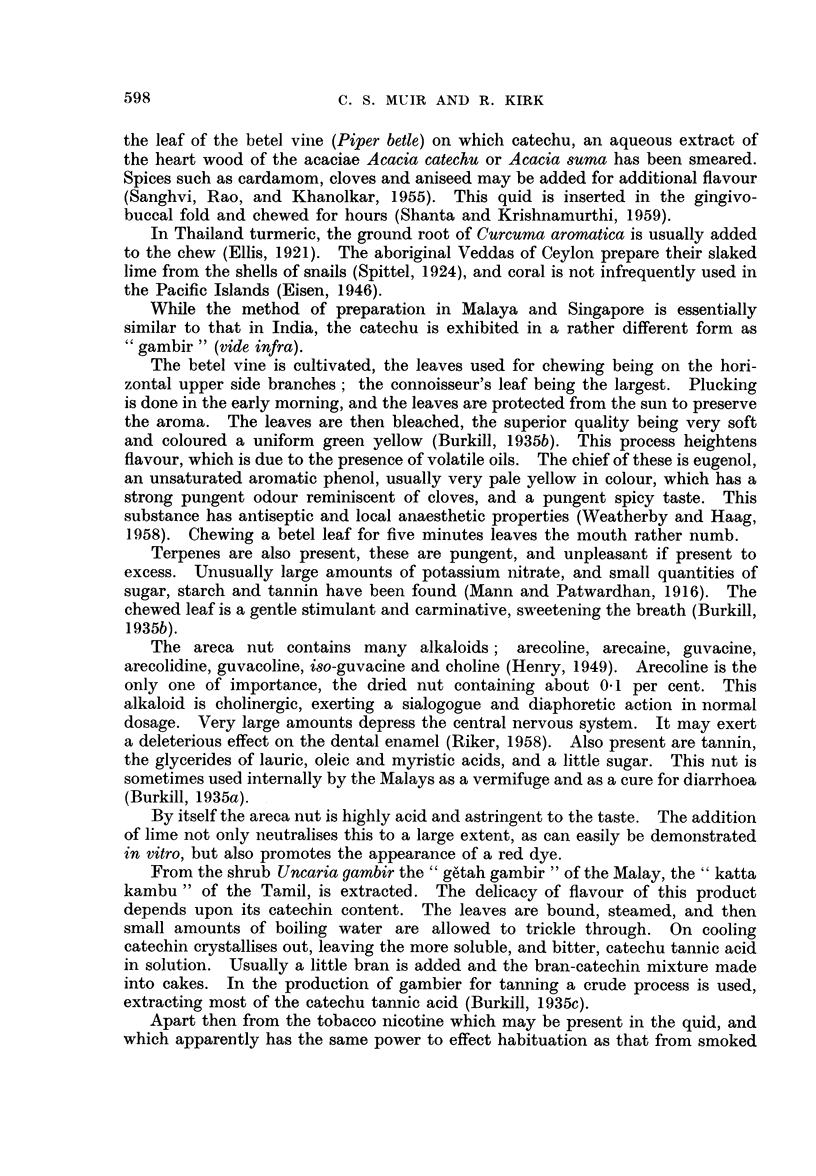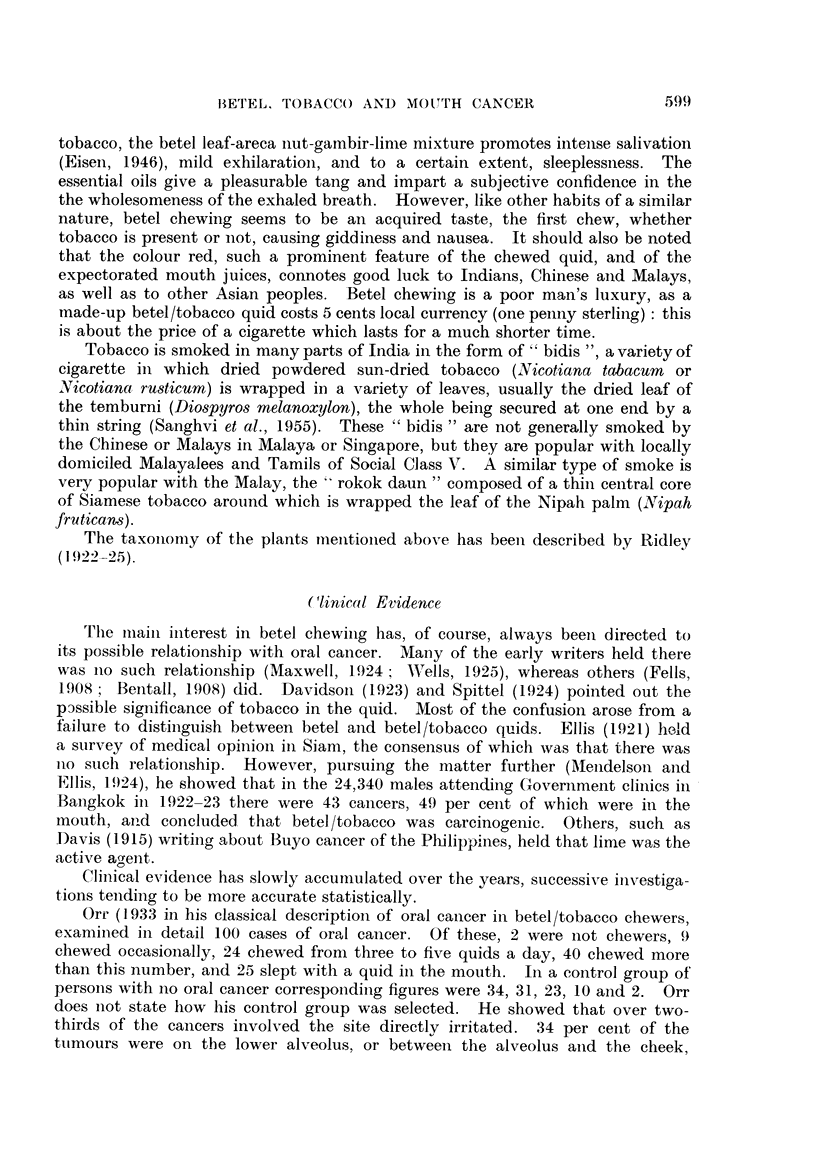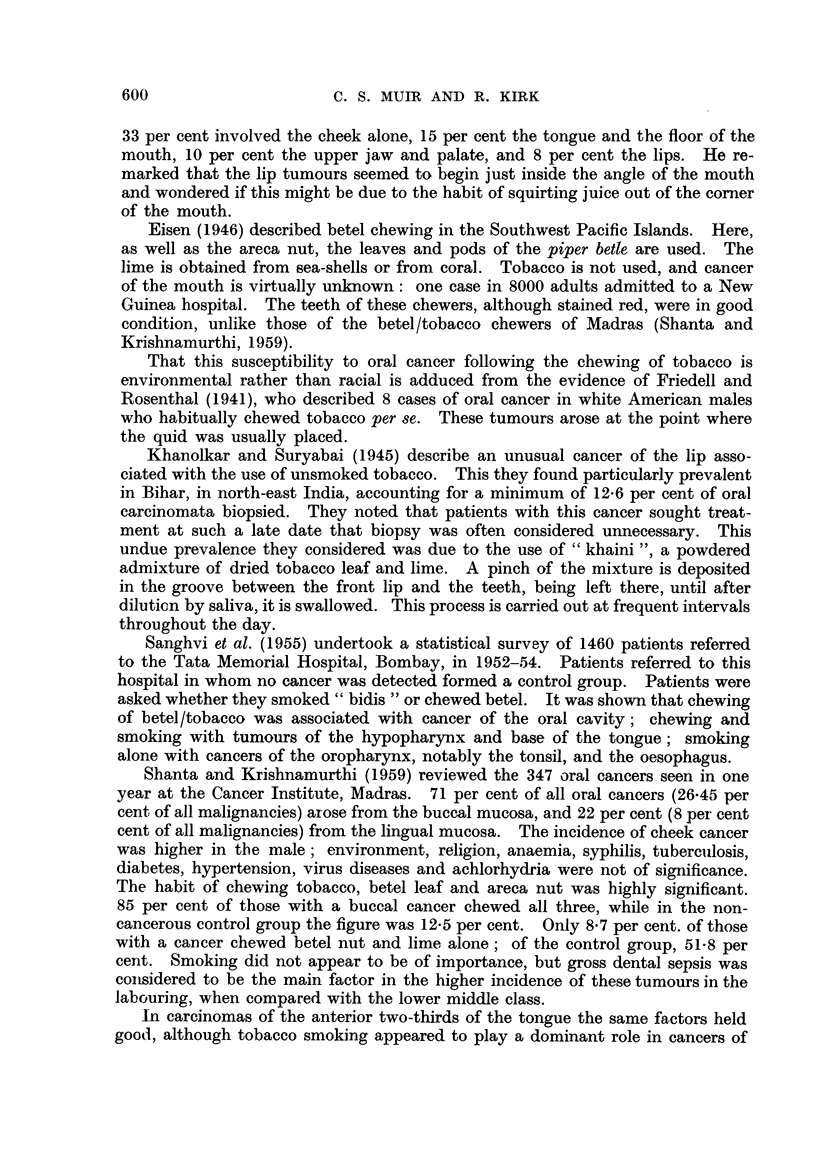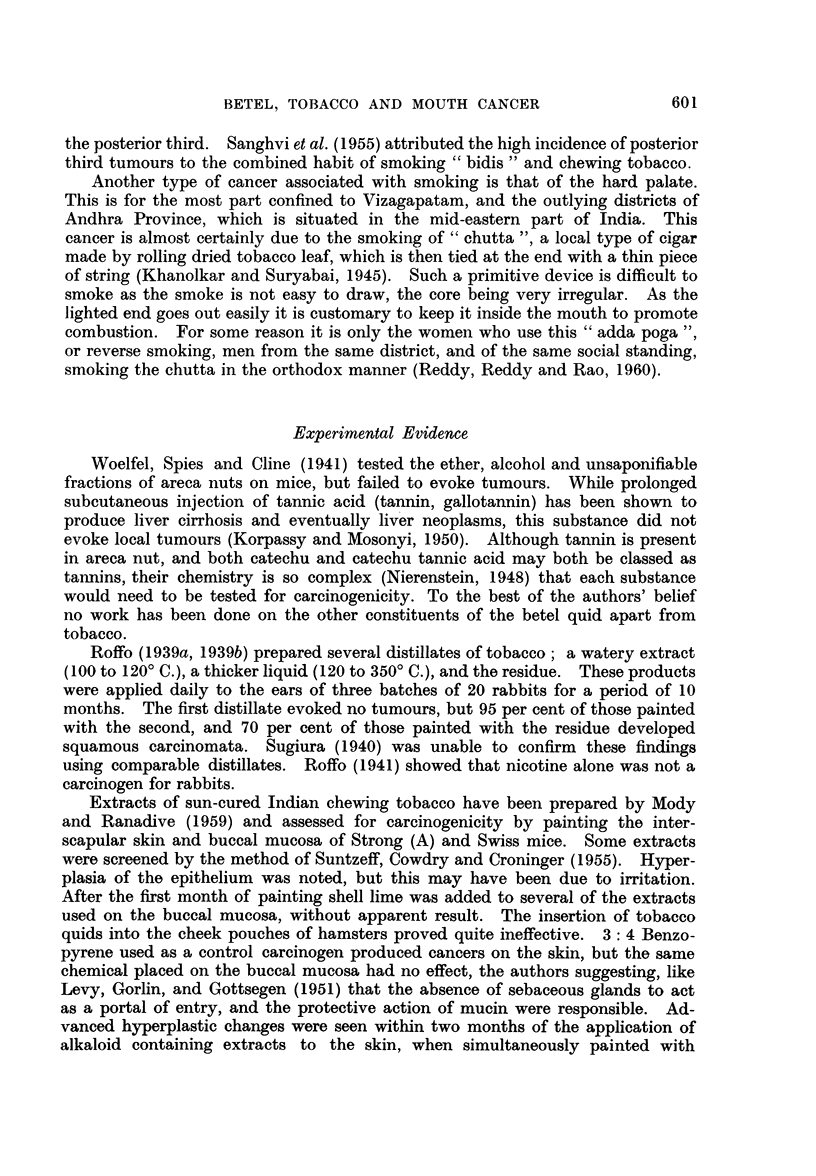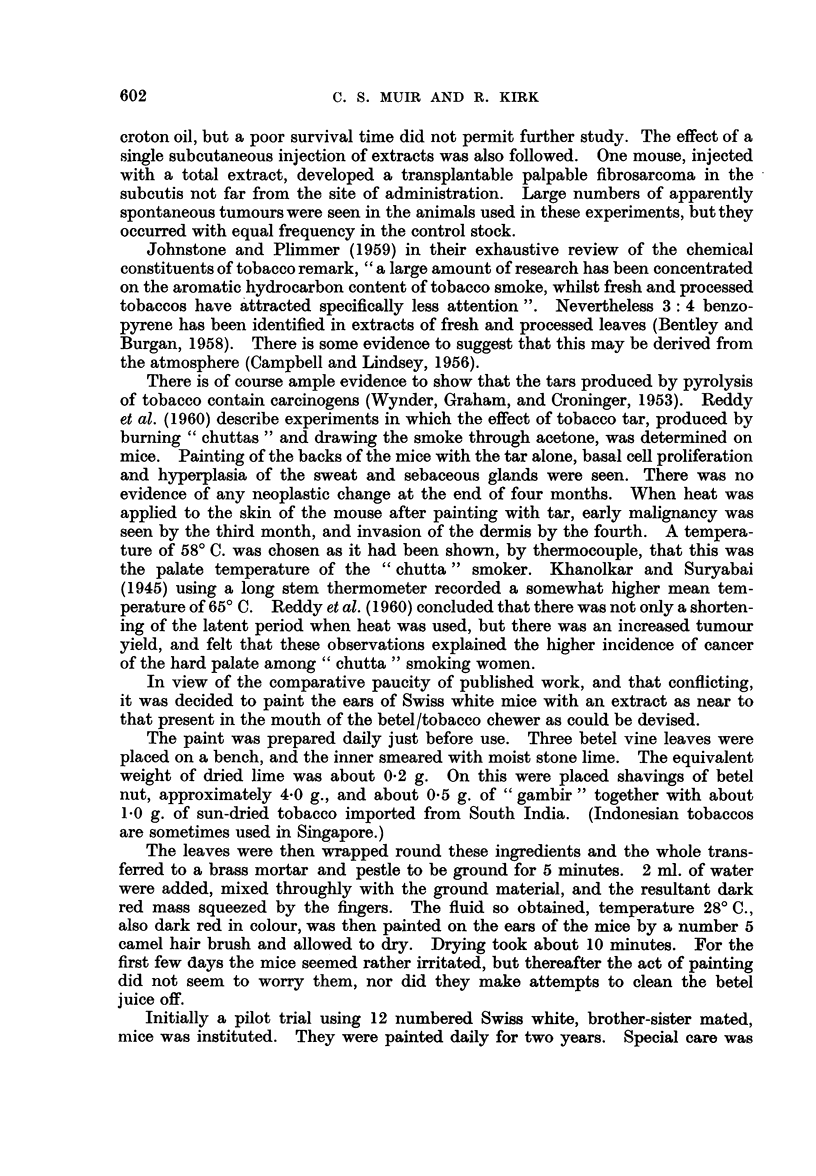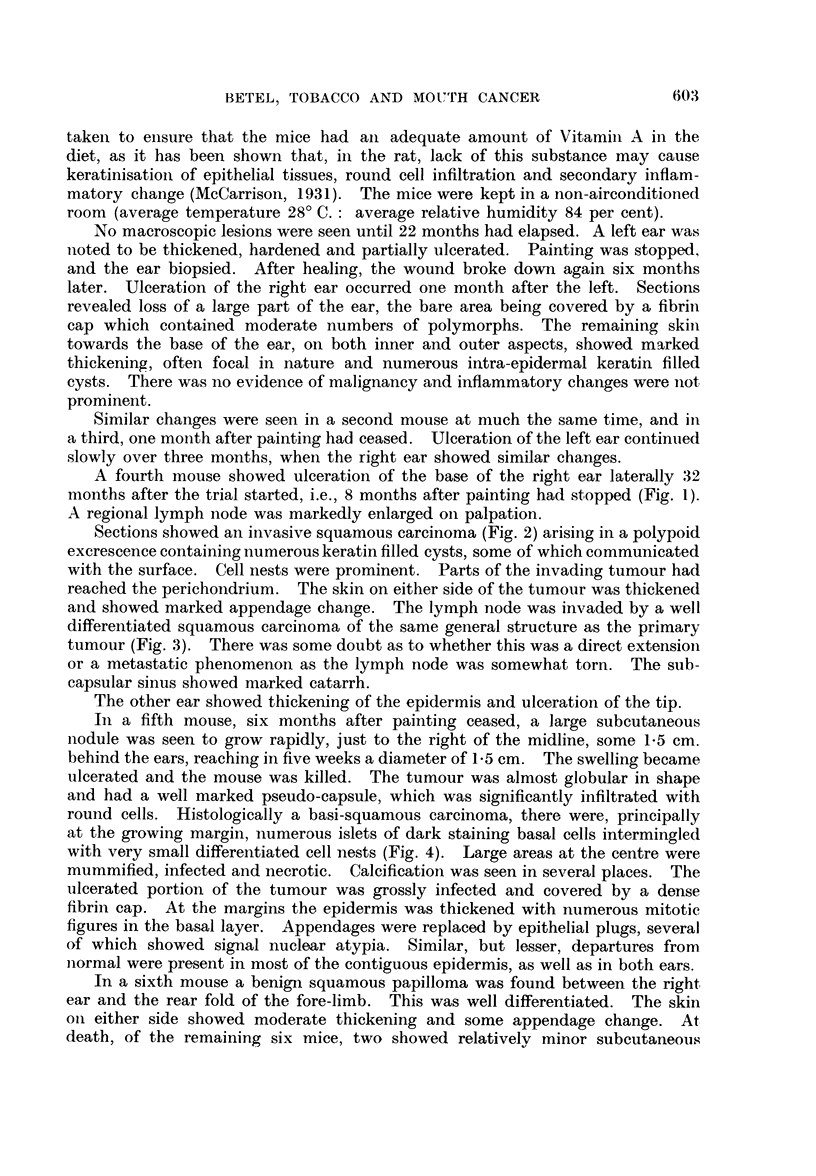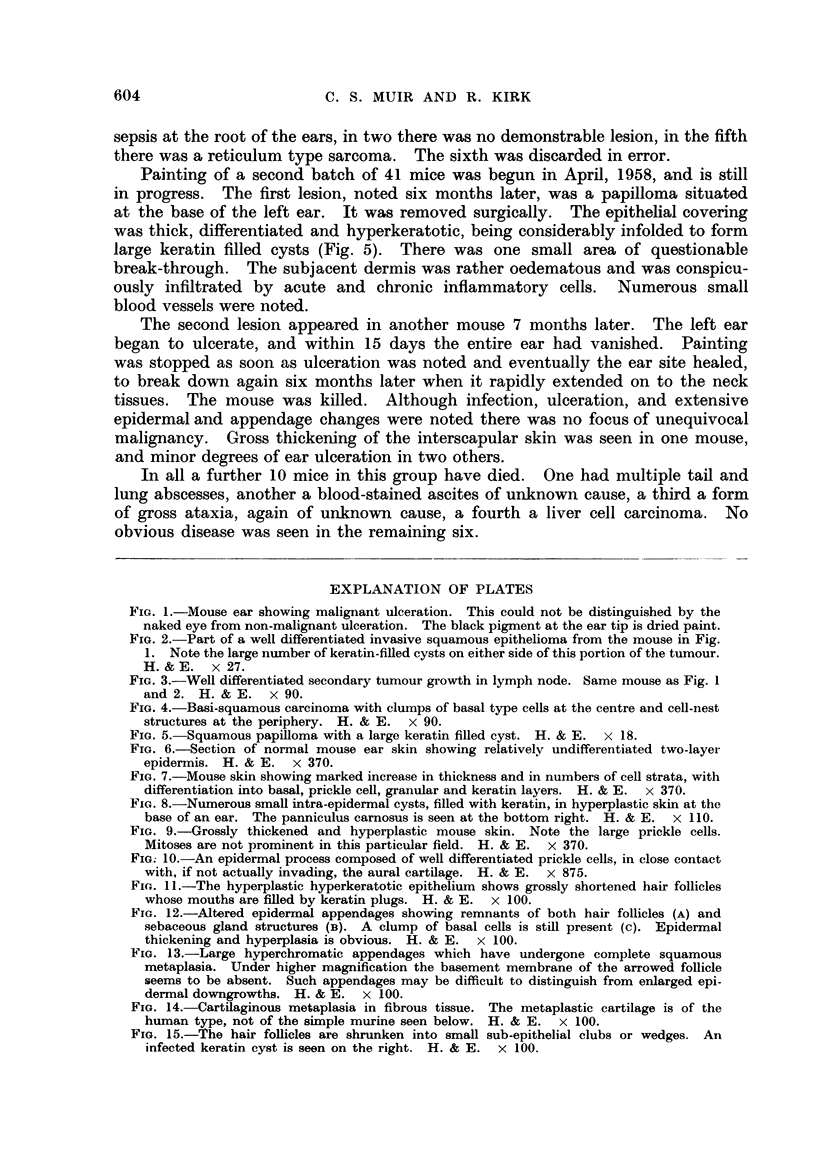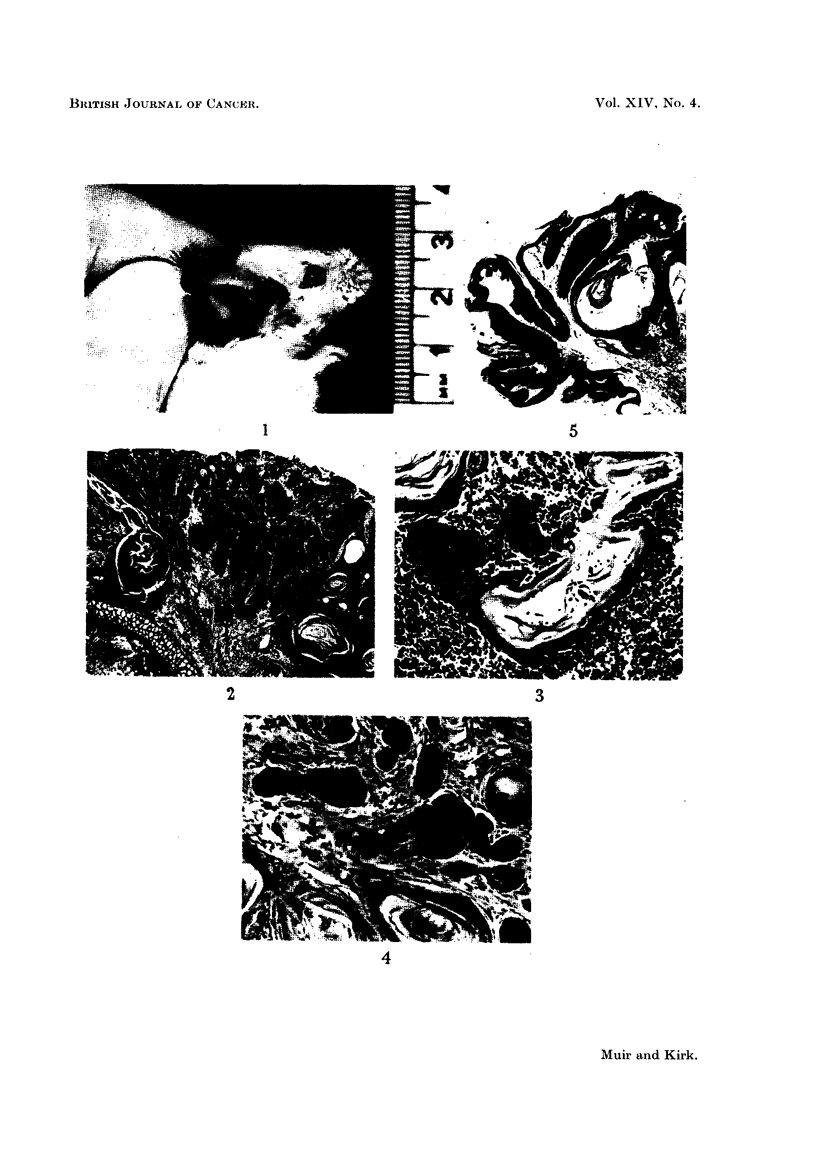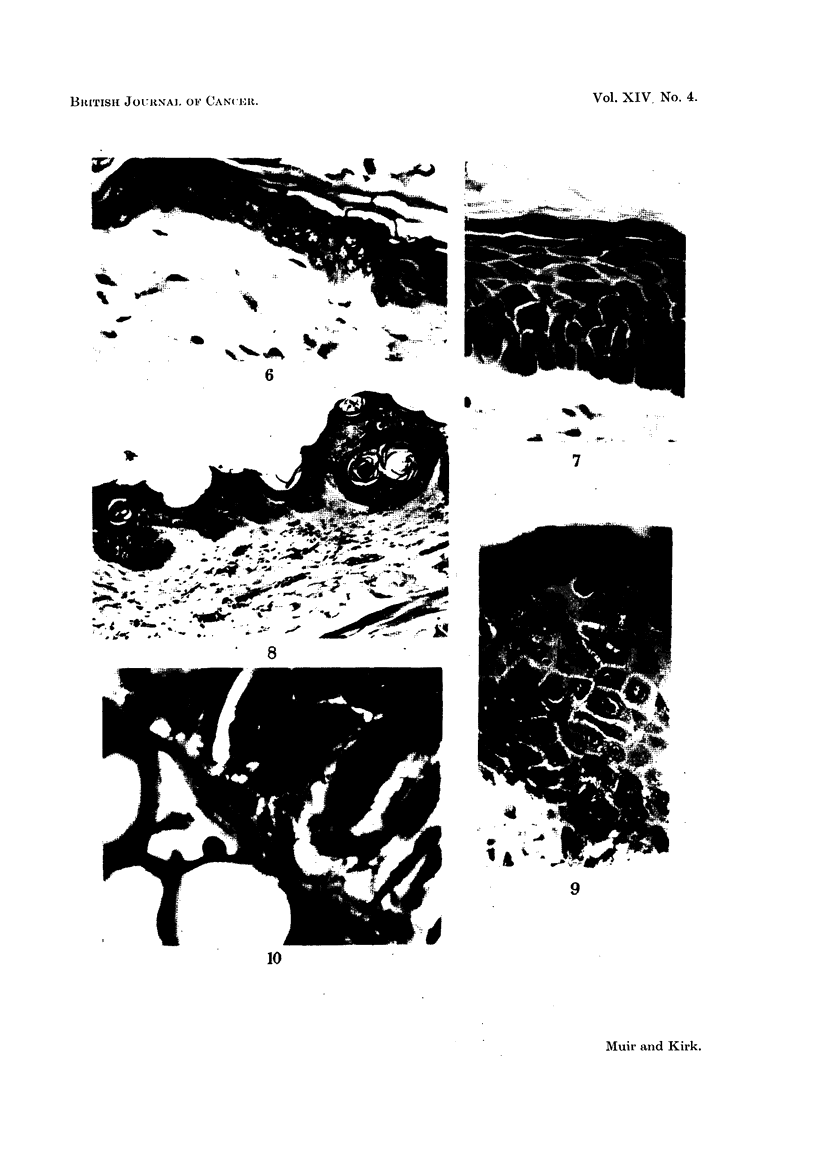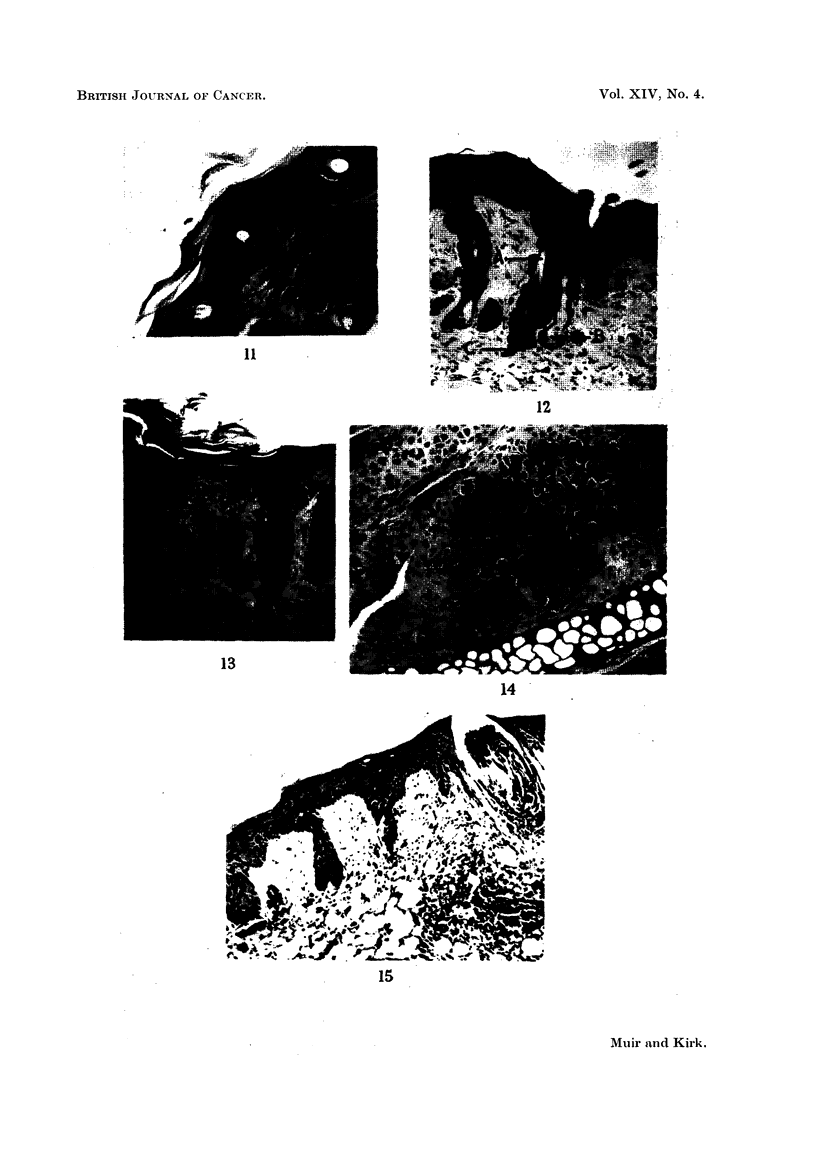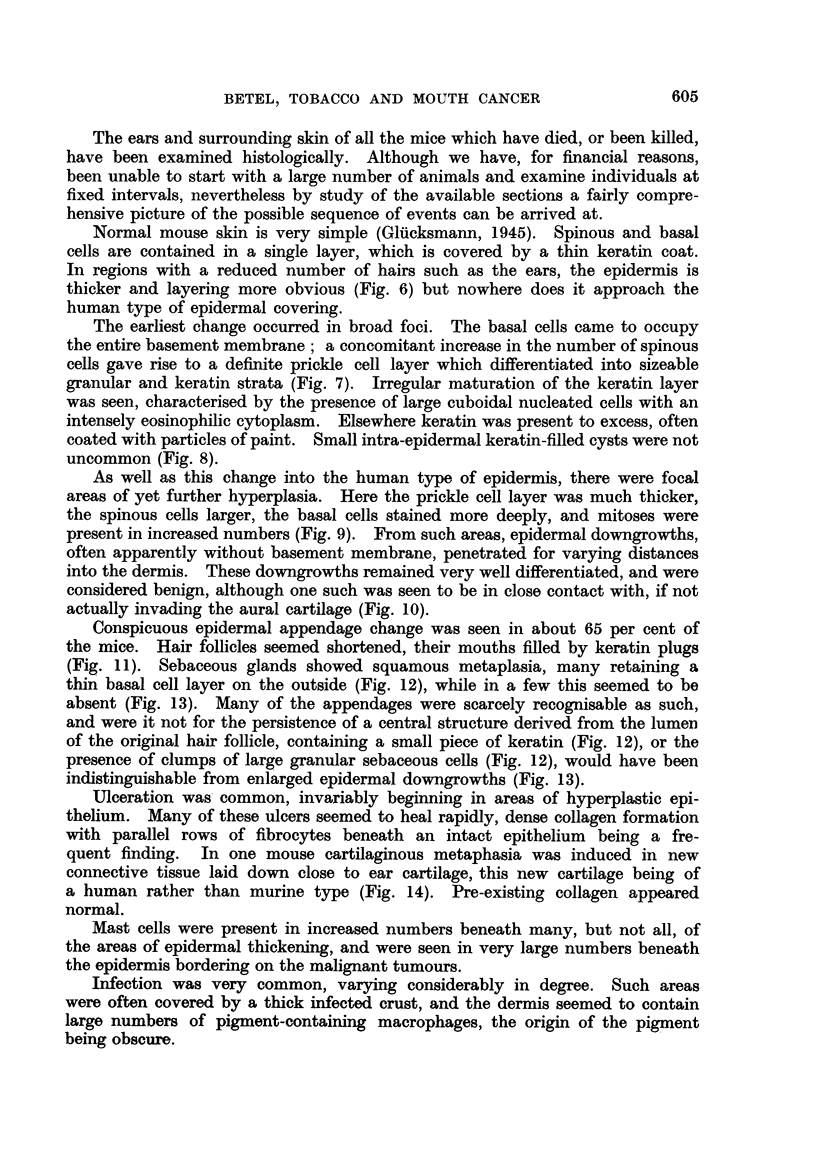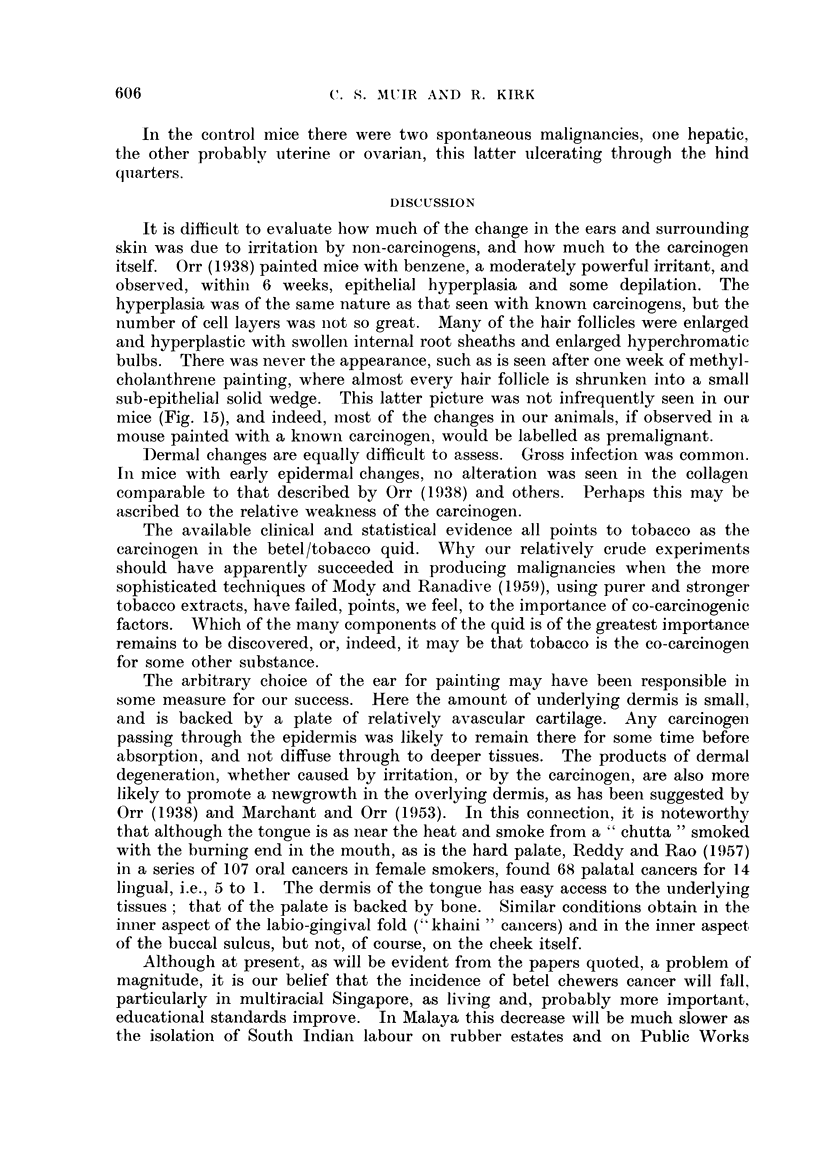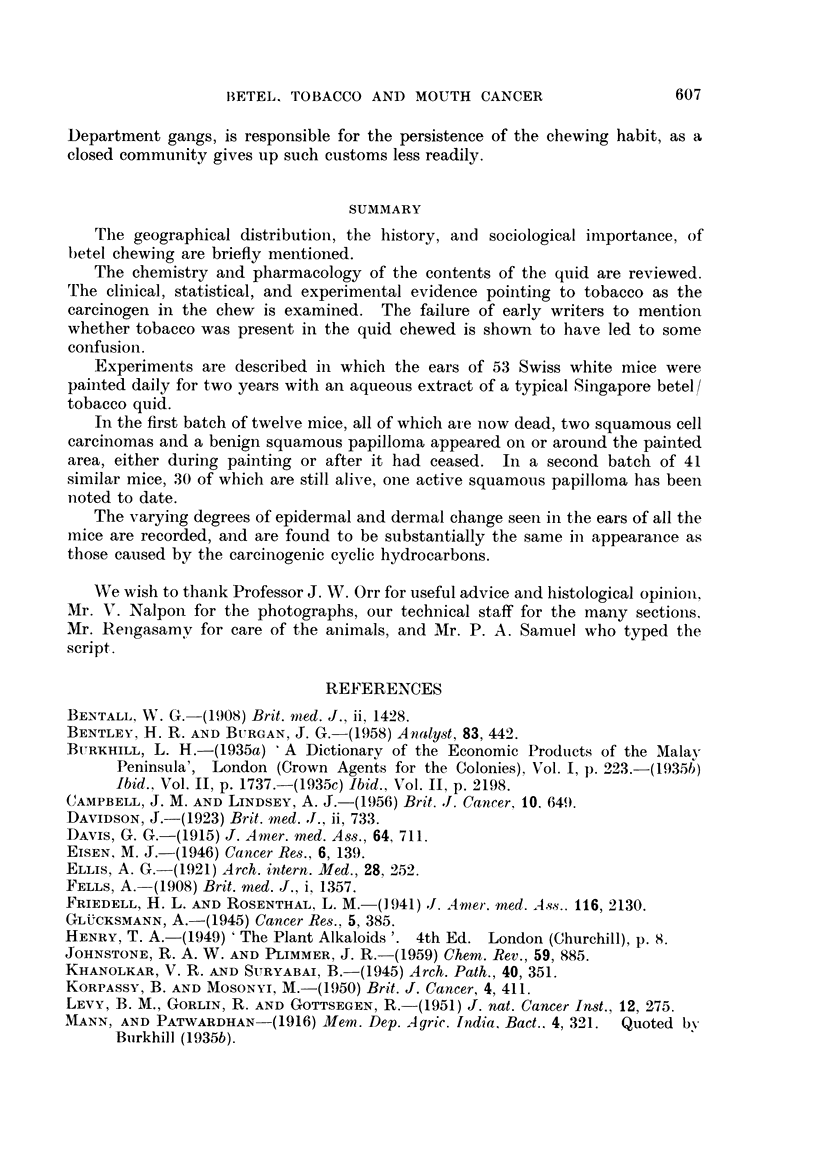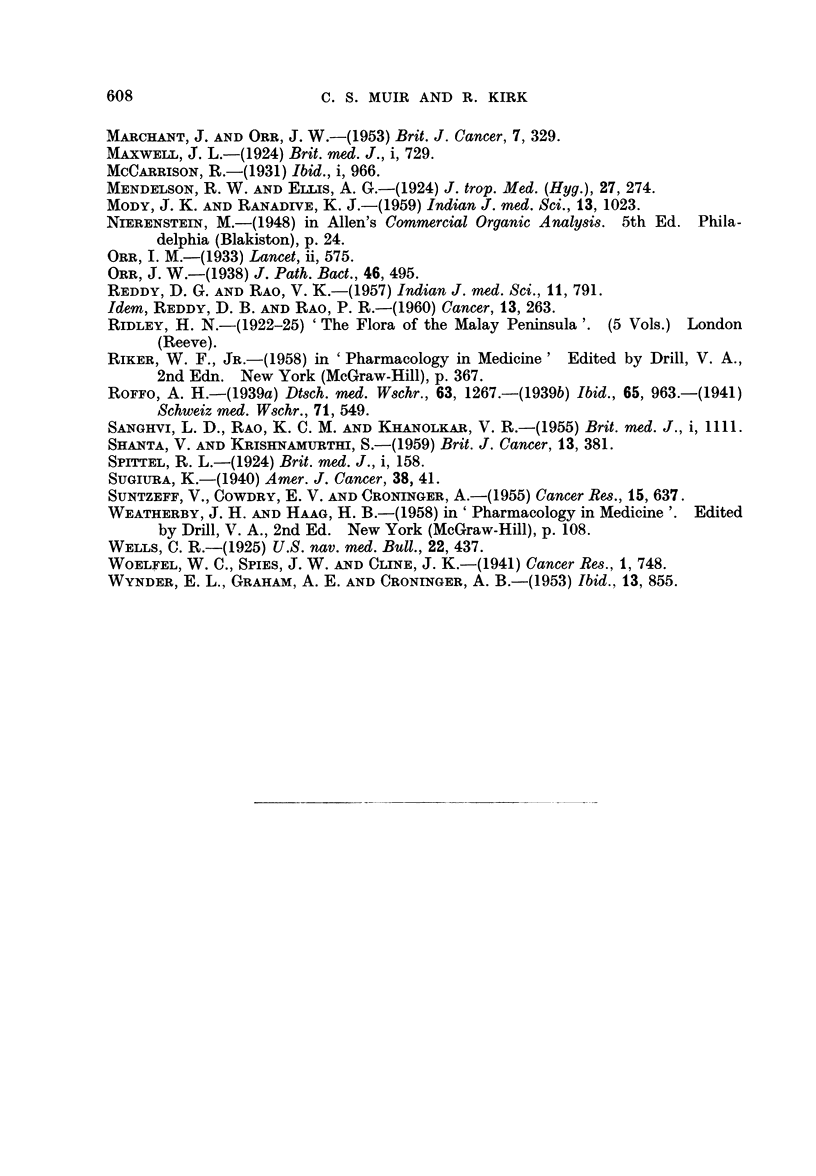# Betel, Tobacco, and Cancer of the Mouth

**DOI:** 10.1038/bjc.1960.65

**Published:** 1960-12

**Authors:** C. S. Muir, R. Kirk

## Abstract

**Images:**


### BRITISH JOURNAL OF CANCER

VOL. XIV DECEMBER, 1960 NO. 4

### BETEL, TOBACCO, AND CANCER OF THE MOUTH

C. S. MUIR AND R. KIRK*

*From the Department of Pathology, University of Malaya in Singapore*

Received for publication August 22, 1960

TOBACCO has been, for many years, under suspicion as a carcinogen. Pride of place has of late been accorded in the literature to the relationship between bronchial carcinoma and cigarette smoking, but in South India and in those parts of South-East Asia where persons of South Indian stock work, or have settled, oral cancer has long been a problem of importance. Essentially there are two types of oral cancer; that associated with the chewing of tobacco, usually admixed with some form of betel (*vide infra*), and that associated with smoked tobacco.

This paper is primarily, but not exclusively, concerned with the former type of tumour, and examines the nature of the betel quid, touches briefly on its complex chemistry and pharmacology, reviews the clinical and experimental evidence pointing to the presence of a carcinogenic agent, probably tobacco, in the quid, and reports experiments in which malignancy was induced in the ears of mice painted for a prolonged period with an aqueous extract of a typical Singapore betel quid.

### *The Quid: Composition, Chemistry, Pharmacology*

The chewing of betel, with or without tobacco, is widespread in the Orient: Ceylon, India, Burma, Siam, Indochina, Malaya, Singapore, Indonesia, the Philippines, New Guinea, New Britain, New Ireland, Formosa and China. The habit is of great antiquity. The chewing of the areca, or betel, nut is mentioned in the Sanskrit " Susrata Samhita " believed to have been written about A.D. 600 near Benares. The Sanskrit for the leaf of the betel vine " tambula ", persists in the modern Hindi " tambuli " and in the Arabic and Persian " tambula ". The Malay " sireh " bears no resemblance, but Malay is not of Sanskrit derivation (Burkill, 1935*b*). The habit is known to have reached the Zanzibar coast between A.D. 1200–1400 and mention is made in Dutch archives of 1664 of an impost duty on betel leaf imported from India into Malacca. In 1703 the import was forbidden, presumably to protect local growers, rather than to prevent a well-established habit.

Tobacco is almost never chewed by itself. In India it is usually mixed, flaked, with cracked, powdered, or sliced dried betel nut (the fruit of the betel palm, *Areca catechu*), and slaked stone or shell lime, the whole being wrapped in

* Present address: Department of Pathology, University of Hongkong.

the leaf of the betel vine (*Piper betle*) on which catechu, an aqueous extract of the heart wood of the acaciae *Acacia catechu* or *Acacia suma* has been smeared. Spices such as cardamom, cloves and aniseed may be added for additional flavour (Sanghvi, Rao, and Khanolkar, 1955). This quid is inserted in the gingivo-buccal fold and chewed for hours (Shanta and Krishnamurthi, 1959).

In Thailand turmeric, the ground root of *Curcuma aromatica* is usually added to the chew (Ellis, 1921). The aboriginal Veddas of Ceylon prepare their slaked lime from the shells of snails (Spittel, 1924), and coral is not infrequently used in the Pacific Islands (Eisen, 1946).

While the method of preparation in Malaya and Singapore is essentially similar to that in India, the catechu is exhibited in a rather different form as " gambir " (*vide infra*).

The betel vine is cultivated, the leaves used for chewing being on the horizontal upper side branches ; the connoisseur's leaf being the largest. Plucking is done in the early morning, and the leaves are protected from the sun to preserve the aroma. The leaves are then bleached, the superior quality being very soft and coloured a uniform green yellow (Burkill, 1935*b*). This process heightens flavour, which is due to the presence of volatile oils. The chief of these is eugenol, an unsaturated aromatic phenol, usually very pale yellow in colour, which has a strong pungent odour reminiscent of cloves, and a pungent spicy taste. This substance has antiseptic and local anaesthetic properties (Weatherby and Haag, 1958). Chewing a betel leaf for five minutes leaves the mouth rather numb.

Terpenes are also present, these are pungent, and unpleasant if present to excess. Unusually large amounts of potassium nitrate, and small quantities of sugar, starch and tannin have been found (Mann and Patwardhan, 1916). The chewed leaf is a gentle stimulant and carminative, sweetening the breath (Burkill, 1935*b*).

The areca nut contains many alkaloids ; arecoline, arecaine, guvacine, arecolidine, guvacoline, *iso*-guvacine and choline (Henry, 1949). Arecoline is the only one of importance, the dried nut containing about 0·1 per cent. This alkaloid is cholinergic, exerting a sialogogue and diaphoretic action in normal dosage. Very large amounts depress the central nervous system. It may exert a deleterious effect on the dental enamel (Riker, 1958). Also present are tannin, the glycerides of lauric, oleic and myristic acids, and a little sugar. This nut is sometimes used internally by the Malays as a vermifuge and as a cure for diarrhoea (Burkill, 1935*a*).

By itself the areca nut is highly acid and astringent to the taste. The addition of lime not only neutralises this to a large extent, as can easily be demonstrated *in vitro*, but also promotes the appearance of a red dye.

From the shrub *Uncaria gambir* the " gĕtah gambir " of the Malay, the " katta kambu " of the Tamil, is extracted. The delicacy of flavour of this product depends upon its catechin content. The leaves are bound, steamed, and then small amounts of boiling water are allowed to trickle through. On cooling catechin crystallises out, leaving the more soluble, and bitter, catechu tannic acid in solution. Usually a little bran is added and the bran-catechin mixture made into cakes. In the production of gambier for tanning a crude process is used, extracting most of the catechu tannic acid (Burkill, 1935*c*).

Apart then from the tobacco nicotine which may be present in the quid, and which apparently has the same power to effect habituation as that from smoked tobacco, the betel leaf-areca nut-gambir-lime mixture promotes intense salivation (Eisen, 1946), mild exhilaration, and to a certain extent, sleeplessness. The essential oils give a pleasurable tang and impart a subjective confidence in the the wholesomeness of the exhaled breath. However, like other habits of a similar nature, betel chewing seems to be an acquired taste, the first chew, whether tobacco is present or not, causing giddiness and nausea. It should also be noted that the colour red, such a prominent feature of the chewed quid, and of the expectorated mouth juices, connotes good luck to Indians, Chinese and Malays, as well as to other Asian peoples. Betel chewing is a poor man's luxury, as a made-up betel/tobacco quid costs 5 cents local currency (one penny sterling) : this is about the price of a cigarette which lasts for a much shorter time.

Tobacco is smoked in many parts of India in the form of " bidis ", a variety of cigarette in which dried powdered sun-dried tobacco (*Nicotiana tabacum* or *Nicotiana rusticum*) is wrapped in a variety of leaves, usually the dried leaf of the temburni (*Diospyros melanoxylon*), the whole being secured at one end by a thin string (Sanghvi *et al.*, 1955). These " bidis " are not generally smoked by the Chinese or Malays in Malaya or Singapore, but they are popular with locally domiciled Malayalees and Tamils of Social Class V. A similar type of smoke is very popular with the Malay, the " rokok daun " composed of a thin central core of Siamese tobacco around which is wrapped the leaf of the Nipah palm (*Nipah fruticans*).

The taxonomy of the plants mentioned above has been described by Ridley (1922–25).

### *Clinical Evidence*

The main interest in betel chewing has, of course, always been directed to its possible relationship with oral cancer. Many of the early writers held there was no such relationship (Maxwell, 1924 ; Wells, 1925), whereas others (Fells, 1908 ; Bentall, 1908) did. Davidson (1923) and Spittel (1924) pointed out the possible significance of tobacco in the quid. Most of the confusion arose from a failure to distinguish between betel and betel/tobacco quids. Ellis (1921) held a survey of medical opinion in Siam, the consensus of which was that there was no such relationship. However, pursuing the matter further (Mendelson and Ellis, 1924), he showed that in the 24,340 males attending Government clinics in Bangkok in 1922–23 there were 43 cancers, 49 per cent of which were in the mouth, and concluded that betel/tobacco was carcinogenic. Others, such as Davis (1915) writing about Buyo cancer of the Philippines, held that lime was the active agent.

Clinical evidence has slowly accumulated over the years, successive investigations tending to be more accurate statistically.

Orr (1933 in his classical description of oral cancer in betel/tobacco chewers, examined in detail 100 cases of oral cancer. Of these, 2 were not chewers, 9 chewed occasionally, 24 chewed from three to five quids a day, 40 chewed more than this number, and 25 slept with a quid in the mouth. In a control group of persons with no oral cancer corresponding figures were 34, 31, 23, 10 and 2. Orr does not state how his control group was selected. He showed that over two-thirds of the cancers involved the site directly irritated. 34 per cent of the tumours were on the lower alveolus, or between the alveolus and the cheek,

33 per cent involved the cheek alone, 15 per cent the tongue and the floor of the mouth, 10 per cent the upper jaw and palate, and 8 per cent the lips. He remarked that the lip tumours seemed to begin just inside the angle of the mouth and wondered if this might be due to the habit of squirting juice out of the corner of the mouth.

Eisen (1946) described betel chewing in the Southwest Pacific Islands. Here, as well as the areca nut, the leaves and pods of the *piper betle* are used. The lime is obtained from sea-shells or from coral. Tobacco is not used, and cancer of the mouth is virtually unknown : one case in 8000 adults admitted to a New Guinea hospital. The teeth of these chewers, although stained red, were in good condition, unlike those of the betel/tobacco chewers of Madras (Shanta and Krishnamurthi, 1959).

That this susceptibility to oral cancer following the chewing of tobacco is environmental rather than racial is adduced from the evidence of Friedell and Rosenthal (1941), who described 8 cases of oral cancer in white American males who habitually chewed tobacco *per se*. These tumours arose at the point where the quid was usually placed.

Khanolkar and Suryabai (1945) describe an unusual cancer of the lip associated with the use of unsmoked tobacco. This they found particularly prevalent in Bihar, in north-east India, accounting for a minimum of 12·6 per cent of oral carcinomata biopsied. They noted that patients with this cancer sought treatment at such a late date that biopsy was often considered unnecessary. This undue prevalence they considered was due to the use of " khaini ", a powdered admixture of dried tobacco leaf and lime. A pinch of the mixture is deposited in the groove between the front lip and the teeth, being left there, until after dilution by saliva, it is swallowed. This process is carried out at frequent intervals throughout the day.

Sanghvi *et al.* (1955) undertook a statistical survey of 1460 patients referred to the Tata Memorial Hospital, Bombay, in 1952–54. Patients referred to this hospital in whom no cancer was detected formed a control group. Patients were asked whether they smoked " bidis " or chewed betel. It was shown that chewing of betel/tobacco was associated with cancer of the oral cavity ; chewing and smoking with tumours of the hypopharynx and base of the tongue ; smoking alone with cancers of the oropharynx, notably the tonsil, and the oesophagus.

Shanta and Krishnamurthi (1959) reviewed the 347 oral cancers seen in one year at the Cancer Institute, Madras. 71 per cent of all oral cancers (26·45 per cent of all malignancies) arose from the buccal mucosa, and 22 per cent (8 per cent cent of all malignancies) from the lingual mucosa. The incidence of cheek cancer was higher in the male ; environment, religion, anaemia, syphilis, tuberculosis, diabetes, hypertension, virus diseases and achlorhydria were not of significance. The habit of chewing tobacco, betel leaf and areca nut was highly significant. 85 per cent of those with a buccal cancer chewed all three, while in the non-cancerous control group the figure was 12·5 per cent. Only 8·7 per cent. of those with a cancer chewed betel nut and lime alone ; of the control group, 51·8 per cent. Smoking did not appear to be of importance, but gross dental sepsis was considered to be the main factor in the higher incidence of these tumours in the labouring, when compared with the lower middle class.

In carcinomas of the anterior two-thirds of the tongue the same factors held good, although tobacco smoking appeared to play a dominant role in cancers of the posterior third. Sanghvi *et al.* (1955) attributed the high incidence of posterior third tumours to the combined habit of smoking " bidis " and chewing tobacco.

Another type of cancer associated with smoking is that of the hard palate. This is for the most part confined to Vizagapatam, and the outlying districts of Andhra Province, which is situated in the mid-eastern part of India. This cancer is almost certainly due to the smoking of " chutta ", a local type of cigar made by rolling dried tobacco leaf, which is then tied at the end with a thin piece of string (Khanolkar and Suryabai, 1945). Such a primitive device is difficult to smoke as the smoke is not easy to draw, the core being very irregular. As the lighted end goes out easily it is customary to keep it inside the mouth to promote combustion. For some reason it is only the women who use this " adda poga ", or reverse smoking, men from the same district, and of the same social standing, smoking the chutta in the orthodox manner (Reddy, Reddy and Rao, 1960).

### *Experimental Evidence*

Woelfel, Spies and Cline (1941) tested the ether, alcohol and unsaponifiable fractions of areca nuts on mice, but failed to evoke tumours. While prolonged subcutaneous injection of tannic acid (tannin, gallotannin) has been shown to produce liver cirrhosis and eventually liver neoplasms, this substance did not evoke local tumours (Korpassy and Mosonyi, 1950). Although tannin is present in areca nut, and both catechu and catechu tannic acid may both be classed as tannins, their chemistry is so complex (Nierenstein, 1948) that each substance would need to be tested for carcinogenicity. To the best of the authors' belief no work has been done on the other constituents of the betel quid apart from tobacco.

Roffo (1939*a*, 1939*b*) prepared several distillates of tobacco ; a watery extract (100 to 120° C.), a thicker liquid (120 to 350° C.), and the residue. These products were applied daily to the ears of three batches of 20 rabbits for a period of 10 months. The first distillate evoked no tumours, but 95 per cent of those painted with the second, and 70 per cent of those painted with the residue developed squamous carcinomata. Sugiura (1940) was unable to confirm these findings using comparable distillates. Roffo (1941) showed that nicotine alone was not a carcinogen for rabbits.

Extracts of sun-cured Indian chewing tobacco have been prepared by Mody and Ranadive (1959) and assessed for carcinogenicity by painting the interscapular skin and buccal mucosa of Strong (A) and Swiss mice. Some extracts were screened by the method of Suntzeff, Cowdry and Croninger (1955). Hyperplasia of the epithelium was noted, but this may have been due to irritation. After the first month of painting shell lime was added to several of the extracts used on the buccal mucosa, without apparent result. The insertion of tobacco quids into the cheek pouches of hamsters proved quite ineffective. 3 : 4 Benzopyrene used as a control carcinogen produced cancers on the skin, but the same chemical placed on the buccal mucosa had no effect, the authors suggesting, like Levy, Gorlin, and Gottsegen (1951) that the absence of sebaceous glands to act as a portal of entry, and the protective action of mucin were responsible. Advanced hyperplastic changes were seen within two months of the application of alkaloid containing extracts to the skin, when simultaneously painted with croton oil, but a poor survival time did not permit further study. The effect of a single subcutaneous injection of extracts was also followed. One mouse, injected with a total extract, developed a transplantable palpable fibrosarcoma in the subcutis not far from the site of administration. Large numbers of apparently spontaneous tumours were seen in the animals used in these experiments, but they occurred with equal frequency in the control stock.

Johnstone and Plimmer (1959) in their exhaustive review of the chemical constituents of tobacco remark, "a large amount of research has been concentrated on the aromatic hydrocarbon content of tobacco smoke, whilst fresh and processed tobaccos have attracted specifically less attention". Nevertheless 3 : 4 benzopyrene has been identified in extracts of fresh and processed leaves (Bentley and Burgan, 1958). There is some evidence to suggest that this may be derived from the atmosphere (Campbell and Lindsey, 1956).

There is of course ample evidence to show that the tars produced by pyrolysis of tobacco contain carcinogens (Wynder, Graham, and Croninger, 1953). Reddy *et al.* (1960) describe experiments in which the effect of tobacco tar, produced by burning "chuttas" and drawing the smoke through acetone, was determined on mice. Painting of the backs of the mice with the tar alone, basal cell proliferation and hyperplasia of the sweat and sebaceous glands were seen. There was no evidence of any neoplastic change at the end of four months. When heat was applied to the skin of the mouse after painting with tar, early malignancy was seen by the third month, and invasion of the dermis by the fourth. A temperature of 58° C. was chosen as it had been shown, by thermocouple, that this was the palate temperature of the "chutta" smoker. Khanolkar and Suryabai (1945) using a long stem thermometer recorded a somewhat higher mean temperature of 65° C. Reddy *et al.* (1960) concluded that there was not only a shortening of the latent period when heat was used, but there was an increased tumour yield, and felt that these observations explained the higher incidence of cancer of the hard palate among "chutta" smoking women.

In view of the comparative paucity of published work, and that conflicting, it was decided to paint the ears of Swiss white mice with an extract as near to that present in the mouth of the betel/tobacco chewer as could be devised.

The paint was prepared daily just before use. Three betel vine leaves were placed on a bench, and the inner smeared with moist stone lime. The equivalent weight of dried lime was about 0·2 g. On this were placed shavings of betel nut, approximately 4·0 g., and about 0·5 g. of "gambir" together with about 1·0 g. of sun-dried tobacco imported from South India. (Indonesian tobaccos are sometimes used in Singapore.)

The leaves were then wrapped round these ingredients and the whole transferred to a brass mortar and pestle to be ground for 5 minutes. 2 ml. of water were added, mixed throughly with the ground material, and the resultant dark red mass squeezed by the fingers. The fluid so obtained, temperature 28° C., also dark red in colour, was then painted on the ears of the mice by a number 5 camel hair brush and allowed to dry. Drying took about 10 minutes. For the first few days the mice seemed rather irritated, but thereafter the act of painting did not seem to worry them, nor did they make attempts to clean the betel juice off.

Initially a pilot trial using 12 numbered Swiss white, brother-sister mated, mice was instituted. They were painted daily for two years. Special care was taken to ensure that the mice had an adequate amount of Vitamin A in the diet, as it has been shown that, in the rat, lack of this substance may cause keratinisation of epithelial tissues, round cell infiltration and secondary inflammatory change (McCarrison, 1931). The mice were kept in a non-airconditioned room (average temperature 28° C. : average relative humidity 84 per cent).

No macroscopic lesions were seen until 22 months had elapsed. A left ear was noted to be thickened, hardened and partially ulcerated. Painting was stopped, and the ear biopsied. After healing, the wound broke down again six months later. Ulceration of the right ear occurred one month after the left. Sections revealed loss of a large part of the ear, the bare area being covered by a fibrin cap which contained moderate numbers of polymorphs. The remaining skin towards the base of the ear, on both inner and outer aspects, showed marked thickening, often focal in nature and numerous intra-epidermal keratin filled cysts. There was no evidence of malignancy and inflammatory changes were not prominent.

Similar changes were seen in a second mouse at much the same time, and in a third, one month after painting had ceased. Ulceration of the left ear continued slowly over three months, when the right ear showed similar changes.

A fourth mouse showed ulceration of the base of the right ear laterally 32 months after the trial started, i.e., 8 months after painting had stopped (Fig. 1). A regional lymph node was markedly enlarged on palpation.

Sections showed an invasive squamous carcinoma (Fig. 2) arising in a polypoid excrescence containing numerous keratin filled cysts, some of which communicated with the surface. Cell nests were prominent. Parts of the invading tumour had reached the perichondrium. The skin on either side of the tumour was thickened and showed marked appendage change. The lymph node was invaded by a well differentiated squamous carcinoma of the same general structure as the primary tumour (Fig. 3). There was some doubt as to whether this was a direct extension or a metastatic phenomenon as the lymph node was somewhat torn. The subcapsular sinus showed marked catarrh.

The other ear showed thickening of the epidermis and ulceration of the tip.

In a fifth mouse, six months after painting ceased, a large subcutaneous nodule was seen to grow rapidly, just to the right of the midline, some 1·5 cm. behind the ears, reaching in five weeks a diameter of 1·5 cm. The swelling became ulcerated and the mouse was killed. The tumour was almost globular in shape and had a well marked pseudo-capsule, which was significantly infiltrated with round cells. Histologically a basi-squamous carcinoma, there were, principally at the growing margin, numerous islets of dark staining basal cells intermingled with very small differentiated cell nests (Fig. 4). Large areas at the centre were mummified, infected and necrotic. Calcification was seen in several places. The ulcerated portion of the tumour was grossly infected and covered by a dense fibrin cap. At the margins the epidermis was thickened with numerous mitotic figures in the basal layer. Appendages were replaced by epithelial plugs, several of which showed signal nuclear atypia. Similar, but lesser, departures from normal were present in most of the contiguous epidermis, as well as in both ears.

In a sixth mouse a benign squamous papilloma was found between the right ear and the rear fold of the fore-limb. This was well differentiated. The skin on either side showed moderate thickening and some appendage change. At death, of the remaining six mice, two showed relatively minor subcutaneous sepsis at the root of the ears, in two there was no demonstrable lesion, in the fifth there was a reticulum type sarcoma. The sixth was discarded in error.

Painting of a second batch of 41 mice was begun in April, 1958, and is still in progress. The first lesion, noted six months later, was a papilloma situated at the base of the left ear. It was removed surgically. The epithelial covering was thick, differentiated and hyperkeratotic, being considerably infolded to form large keratin filled cysts (Fig. 5). There was one small area of questionable break-through. The subjacent dermis was rather oedematous and was conspicuously infiltrated by acute and chronic inflammatory cells. Numerous small blood vessels were noted.

The second lesion appeared in another mouse 7 months later. The left ear began to ulcerate, and within 15 days the entire ear had vanished. Painting was stopped as soon as ulceration was noted and eventually the ear site healed, to break down again six months later when it rapidly extended on to the neck tissues. The mouse was killed. Although infection, ulceration, and extensive epidermal and appendage changes were noted there was no focus of unequivocal malignancy. Gross thickening of the interscapular skin was seen in one mouse, and minor degrees of ear ulceration in two others.

In all a further 10 mice in this group have died. One had multiple tail and lung abscesses, another a blood-stained ascites of unknown cause, a third a form of gross ataxia, again of unknown cause, a fourth a liver cell carcinoma. No obvious disease was seen in the remaining six.

---

### EXPLANATION OF PLATES

FIG. 1.—Mouse ear showing malignant ulceration. This could not be distinguished by the naked eye from non-malignant ulceration. The black pigment at the ear tip is dried paint.

FIG. 2.—Part of a well differentiated invasive squamous epithelioma from the mouse in Fig. 1. Note the large number of keratin-filled cysts on either side of this portion of the tumour. H. & E. × 27.

FIG. 3.—Well differentiated secondary tumour growth in lymph node. Same mouse as Fig. 1 and 2. H. & E. × 90.

FIG. 4.—Basi-squamous carcinoma with clumps of basal type cells at the centre and cell-nest structures at the periphery. H. & E. × 90.

FIG. 5.—Squamous papilloma with a large keratin filled cyst. H. & E. × 18.

FIG. 6.—Section of normal mouse ear skin showing relatively undifferentiated two-layer epidermis. H. & E. × 370.

FIG. 7.—Mouse skin showing marked increase in thickness and in numbers of cell strata, with differentiation into basal, prickle cell, granular and keratin layers. H. & E. × 370.

FIG. 8.—Numerous small intra-epidermal cysts, filled with keratin, in hyperplastic skin at the base of an ear. The panniculus carnosus is seen at the bottom right. H. & E. × 110.

FIG. 9.—Grossly thickened and hyperplastic mouse skin. Note the large prickle cells. Mitoses are not prominent in this particular field. H. & E. × 370.

FIG. 10.—An epidermal process composed of well differentiated prickle cells, in close contact with, if not actually invading, the aural cartilage. H. & E. × 875.

FIG. 11.—The hyperplastic hyperkeratotic epithelium shows grossly shortened hair follicles whose mouths are filled by keratin plugs. H. & E. × 100.

FIG. 12.—Altered epidermal appendages showing remnants of both hair follicles (A) and sebaceous gland structures (B). A clump of basal cells is still present (C). Epidermal thickening and hyperplasia is obvious. H. & E. × 100.

FIG. 13.—Large hyperchromatic appendages which have undergone complete squamous metaplasia. Under higher magnification the basement membrane of the arrowed follicle seems to be absent. Such appendages may be difficult to distinguish from enlarged epidermal downgrowths. H. & E. × 100.

FIG. 14.—Cartilaginous metaplasia in fibrous tissue. The metaplastic cartilage is of the human type, not of the simple murine seen below. H. & E. × 100.

FIG. 15.—The hair follicles are shrunken into small sub-epithelial clubs or wedges. An infected keratin cyst is seen on the right. H. & E. × 100.

1

5

2

3

4

6

7

8

9

10

11

12

13

14

15

The ears and surrounding skin of all the mice which have died, or been killed, have been examined histologically. Although we have, for financial reasons, been unable to start with a large number of animals and examine individuals at fixed intervals, nevertheless by study of the available sections a fairly comprehensive picture of the possible sequence of events can be arrived at.

Normal mouse skin is very simple (Glücksmann, 1945). Spinous and basal cells are contained in a single layer, which is covered by a thin keratin coat. In regions with a reduced number of hairs such as the ears, the epidermis is thicker and layering more obvious (Fig. 6) but nowhere does it approach the human type of epidermal covering.

The earliest change occurred in broad foci. The basal cells came to occupy the entire basement membrane ; a concomitant increase in the number of spinous cells gave rise to a definite prickle cell layer which differentiated into sizeable granular and keratin strata (Fig. 7). Irregular maturation of the keratin layer was seen, characterised by the presence of large cuboidal nucleated cells with an intensely eosinophilic cytoplasm. Elsewhere keratin was present to excess, often coated with particles of paint. Small intra-epidermal keratin-filled cysts were not uncommon (Fig. 8).

As well as this change into the human type of epidermis, there were focal areas of yet further hyperplasia. Here the prickle cell layer was much thicker, the spinous cells larger, the basal cells stained more deeply, and mitoses were present in increased numbers (Fig. 9). From such areas, epidermal downgrowths, often apparently without basement membrane, penetrated for varying distances into the dermis. These downgrowths remained very well differentiated, and were considered benign, although one such was seen to be in close contact with, if not actually invading the aural cartilage (Fig. 10).

Conspicuous epidermal appendage change was seen in about 65 per cent of the mice. Hair follicles seemed shortened, their mouths filled by keratin plugs (Fig. 11). Sebaceous glands showed squamous metaplasia, many retaining a thin basal cell layer on the outside (Fig. 12), while in a few this seemed to be absent (Fig. 13). Many of the appendages were scarcely recognisable as such, and were it not for the persistence of a central structure derived from the lumen of the original hair follicle, containing a small piece of keratin (Fig. 12), or the presence of clumps of large granular sebaceous cells (Fig. 12), would have been indistinguishable from enlarged epidermal downgrowths (Fig. 13).

Ulceration was common, invariably beginning in areas of hyperplastic epithelium. Many of these ulcers seemed to heal rapidly, dense collagen formation with parallel rows of fibrocytes beneath an intact epithelium being a frequent finding. In one mouse cartilaginous metaphasia was induced in new connective tissue laid down close to ear cartilage, this new cartilage being of a human rather than murine type (Fig. 14). Pre-existing collagen appeared normal.

Mast cells were present in increased numbers beneath many, but not all, of the areas of epidermal thickening, and were seen in very large numbers beneath the epidermis bordering on the malignant tumours.

Infection was very common, varying considerably in degree. Such areas were often covered by a thick infected crust, and the dermis seemed to contain large numbers of pigment-containing macrophages, the origin of the pigment being obscure.

In the control mice there were two spontaneous malignancies, one hepatic, the other probably uterine or ovarian, this latter ulcerating through the hind quarters.

### DISCUSSION

It is difficult to evaluate how much of the change in the ears and surrounding skin was due to irritation by non-carcinogens, and how much to the carcinogen itself. Orr (1938) painted mice with benzene, a moderately powerful irritant, and observed, within 6 weeks, epithelial hyperplasia and some depilation. The hyperplasia was of the same nature as that seen with known carcinogens, but the number of cell layers was not so great. Many of the hair follicles were enlarged and hyperplastic with swollen internal root sheaths and enlarged hyperchromatic bulbs. There was never the appearance, such as is seen after one week of methylcholanthrene painting, where almost every hair follicle is shrunken into a small sub-epithelial solid wedge. This latter picture was not infrequently seen in our mice (Fig. 15), and indeed, most of the changes in our animals, if observed in a mouse painted with a known carcinogen, would be labelled as premalignant.

Dermal changes are equally difficult to assess. Gross infection was common. In mice with early epidermal changes, no alteration was seen in the collagen comparable to that described by Orr (1938) and others. Perhaps this may be ascribed to the relative weakness of the carcinogen.

The available clinical and statistical evidence all points to tobacco as the carcinogen in the betel/tobacco quid. Why our relatively crude experiments should have apparently succeeded in producing malignancies when the more sophisticated techniques of Mody and Ranadive (1959), using purer and stronger tobacco extracts, have failed, points, we feel, to the importance of co-carcinogenic factors. Which of the many components of the quid is of the greatest importance remains to be discovered, or, indeed, it may be that tobacco is the co-carcinogen for some other substance.

The arbitrary choice of the ear for painting may have been responsible in some measure for our success. Here the amount of underlying dermis is small, and is backed by a plate of relatively avascular cartilage. Any carcinogen passing through the epidermis was likely to remain there for some time before absorption, and not diffuse through to deeper tissues. The products of dermal degeneration, whether caused by irritation, or by the carcinogen, are also more likely to promote a newgrowth in the overlying dermis, as has been suggested by Orr (1938) and Marchant and Orr (1953). In this connection, it is noteworthy that although the tongue is as near the heat and smoke from a " chutta " smoked with the burning end in the mouth, as is the hard palate, Reddy and Rao (1957) in a series of 107 oral cancers in female smokers, found 68 palatal cancers for 14 lingual, i.e., 5 to 1. The dermis of the tongue has easy access to the underlying tissues ; that of the palate is backed by bone. Similar conditions obtain in the inner aspect of the labio-gingival fold ("khaini " cancers) and in the inner aspect of the buccal sulcus, but not, of course, on the cheek itself.

Although at present, as will be evident from the papers quoted, a problem of magnitude, it is our belief that the incidence of betel chewers cancer will fall, particularly in multiracial Singapore, as living and, probably more important, educational standards improve. In Malaya this decrease will be much slower as the isolation of South Indian labour on rubber estates and on Public Works

Department gangs, is responsible for the persistence of the chewing habit, as a closed community gives up such customs less readily.

SUMMARY

The geographical distribution, the history, and sociological importance, of betel chewing are briefly mentioned.

The chemistry and pharmacology of the contents of the quid are reviewed. The clinical, statistical, and experimental evidence pointing to tobacco as the carcinogen in the chew is examined. The failure of early writers to mention whether tobacco was present in the quid chewed is shown to have led to some confusion.

Experiments are described in which the ears of 53 Swiss white mice were painted daily for two years with an aqueous extract of a typical Singapore betel/tobacco quid.

In the first batch of twelve mice, all of which are now dead, two squamous cell carcinomas and a benign squamous papilloma appeared on or around the painted area, either during painting or after it had ceased. In a second batch of 41 similar mice, 30 of which are still alive, one active squamous papilloma has been noted to date.

The varying degrees of epidermal and dermal change seen in the ears of all the mice are recorded, and are found to be substantially the same in appearance as those caused by the carcinogenic cyclic hydrocarbons.

We wish to thank Professor J. W. Orr for useful advice and histological opinion, Mr. V. Nalpon for the photographs, our technical staff for the many sections, Mr. Rengasamy for care of the animals, and Mr. P. A. Samuel who typed the script.

REFERENCES

BENTALL, W. G.—(1908) *Brit. med. J.*, ii, 1428.
BENTLEY, H. R. AND BURGAN, J. G.—(1958) *Analyst*, **83**, 442.
BURKHILL, L. H.—(1935*a*) ' A Dictionary of the Economic Products of the Malay Peninsula', London (Crown Agents for the Colonies), Vol. I, p. 223.—(1935*b*) *Ibid.*, Vol. II, p. 1737.—(1935*c*) *Ibid.*, Vol. II, p. 2198.
CAMPBELL, J. M. AND LINDSEY, A. J.—(1956) *Brit. J. Cancer*, **10**, 649.
DAVIDSON, J.—(1923) *Brit. med. J.*, ii, 733.
DAVIS, G. G.—(1915) *J. Amer. med. Ass.*, **64**, 711.
EISEN, M. J.—(1946) *Cancer Res.*, **6**, 139.
ELLIS, A. G.—(1921) *Arch. intern. Med.*, **28**, 252.
FELLS, A.—(1908) *Brit. med. J.*, i, 1357.
FRIEDELL, H. L. AND ROSENTHAL, L. M.—(1941) *J. Amer. med. Ass.*, **116**, 2130.
GLÜCKSMANN, A.—(1945) *Cancer Res.*, **5**, 385.
HENRY, T. A.—(1949) ' The Plant Alkaloids '. 4th Ed. London (Churchill), p. 8.
JOHNSTONE, R. A. W. AND PLIMMER, J. R.—(1959) *Chem. Rev.*, **59**, 885.
KHANOLKAR, V. R. AND SURYABAI, B.—(1945) *Arch. Path.*, **40**, 351.
KORPASSY, B. AND MOSONYI, M.—(1950) *Brit. J. Cancer*, **4**, 411.
LEVY, B. M., GORLIN, R. AND GOTTSEGEN, R.—(1951) *J. nat. Cancer Inst.*, **12**, 275.
MANN, AND PATWARDHAN—(1916) *Mem. Dep. Agric. India, Bact.*, **4**, 321. Quoted by Burkhill (1935*b*).

MARCHANT, J. AND ORR, J. W.—(1953) *Brit. J. Cancer*, **7**, 329.
MAXWELL, J. L.—(1924) *Brit. med. J.*, i, 729.
MCCARRISON, R.—(1931) *Ibid.*, i, 966.
MENDELSON, R. W. AND ELLIS, A. G.—(1924) *J. trop. Med.* (*Hyg.*), **27**, 274.
MODY, J. K. AND RANADIVE, K. J.—(1959) *Indian J. med. Sci.*, **13**, 1023.
NIERENSTEIN, M.—(1948) in Allen's *Commercial Organic Analysis.* 5th Ed. Philadelphia (Blakiston), p. 24.
ORR, I. M.—(1933) *Lancet*, ii, 575.
ORR, J. W.—(1938) *J. Path. Bact.*, **46**, 495.
REDDY, D. G. AND RAO, V. K.—(1957) *Indian J. med. Sci.*, **11**, 791.
*Idem*, REDDY, D. B. AND RAO, P. R.—(1960) *Cancer*, **13**, 263.
RIDLEY, H. N.—(1922–25) ' The Flora of the Malay Peninsula '. (5 Vols.) London (Reeve).
RIKER, W. F., JR.—(1958) in ' Pharmacology in Medicine ' Edited by Drill, V. A., 2nd Edn. New York (McGraw-Hill), p. 367.
ROFFO, A. H.—(1939*a*) *Dtsch. med. Wschr.*, **63**, 1267.—(1939*b*) *Ibid.*, **65**, 963.—(1941) *Schweiz med. Wschr.*, **71**, 549.
SANGHVI, L. D., RAO, K. C. M. AND KHANOLKAR, V. R.—(1955) *Brit. med. J.*, i, 1111.
SHANTA, V. AND KRISHNAMURTHI, S.—(1959) *Brit. J. Cancer*, **13**, 381.
SPITTEL, R. L.—(1924) *Brit. med. J.*, i, 158.
SUGIURA, K.—(1940) *Amer. J. Cancer*, **38**, 41.
SUNTZEFF, V., COWDRY, E. V. AND CRONINGER, A.—(1955) *Cancer Res.*, **15**, 637.
WEATHERBY, J. H. AND HAAG, H. B.—(1958) in ' Pharmacology in Medicine '. Edited by Drill, V. A., 2nd Ed. New York (McGraw-Hill), p. 108.
WELLS, C. R.—(1925) *U.S. nav. med. Bull.*, **22**, 437.
WOELFEL, W. C., SPIES, J. W. AND CLINE, J. K.—(1941) *Cancer Res.*, **1**, 748.
WYNDER, E. L., GRAHAM, A. E. AND CRONINGER, A. B.—(1953) *Ibid.*, **13**, 855.